# Analysis of Whole-Body Coordination Patterning in Successful and Faulty Spikes Using Self-Organising Map-Based Cluster Analysis: A Secondary Analysis

**DOI:** 10.3390/s21041345

**Published:** 2021-02-14

**Authors:** Javad Sarvestan, Zdeněk Svoboda, Fatemeh Alaei, Franky Mulloy

**Affiliations:** 1 Department of Natural Sciences in Kinanthropology, Faculty of Physical Culture, Palacky University Olomouc, 779 00 Olomouc, Czech Republic; Zdenek.svoboda@upol.cz (Z.S.); Fatemeh.alaei01@upol.cz (F.A.); 2 School of Sport and Exercise Science, University of Lincoln, Lincolnshire LN6 7TS, UK; FMulloy@lincoln.ac.uk

**Keywords:** SOM, angular velocity, coordination, volleyball spike, unsupervised machine learning

## Abstract

This study investigated the whole-body coordination patterning in successful and faulty spikes using self-organising map-based cluster analysis. Ten young, elite volleyball players (aged 15.5 ± 0.7 years) performed 60 volleyball spikes in a real-game environment. Adopting the cluster analysis, based on a self-organising map, whole-body coordination patterning was explored between successful and faulty spikes of individual players. The self-organising maps (SOMs) portrayed whole body, lower and upper limb coordination dissimilarities during the jump phase and the ball impact phases between the successful and faulty spikes. The cluster analysis illustrated that the whole body, upper limb and lower limb coordination patterning of each individual’s successful spikes were similar to their faulty spikes. Range of motion patterning also demonstrated no differences in kinematics between spike outcomes. Further, the upper limb angular velocity patterning of the players’ successful/faulty spikes were similar. The SPM analysis portrayed significant differences between the normalized upper limb angular velocities from 35% to 45% and from 76% to 100% of the spike movement. Although the lower limb angular velocities are vital for achieving higher jumps in volleyball spikes, the results of this study portrayed that the upper limb angular velocities distinguish the differences between successful and faulty spikes among the attackers. This confirms the fact that volleyball coaches should shift their focus toward the upper limb velocity and coordination training for higher success rates in spiking for volleyball attackers.

## 1. Introduction

Success in volleyball competitions is directly related to the attacking capabilities of the offensive players [1]. Spikes, as the second and the most important attacking tool, play a pivotal role in this success rate. A successful attack demands highly coordinated actions of the neuromuscular system [2]. Various physical (e.g., strength, coordination) and psychological attributes (e.g., game pressure, scores) underpin success rates in the volleyball spike [3,4,5]. Kinematic analysis of successful spike performance has highlighted movement patterns that result in a faulty spike [1]. Nevertheless, a more sophisticated analysis assessing multiple segment interaction could pave the way for a better understanding of underlying mechanisms that contribute to a better spike performance, particularly during competition [1,6]. 

Several studies have biomechanically investigated the volleyball spike among elite and sub-elite male and female volleyball attackers [2,7,8,9,10]. Wagner, Tilp [11] endorsed that approach velocity, knee angle, and arm swing are principal factors for a more effective spike performance. Serrien, Ooijen [12] highlighted a significant difference between trunk lateral and sagittal tilt, and rotational velocities, pelvis sagittal tilt velocities, and shoulder horizontal abduction and internal rotation velocities between elite and sub-elite male and sub-elite female volleyball players. Fuchs, Menzel [6] demonstrated that jump height, approach speed, step length, mean lower limb muscle activation, and net impulse are significantly higher in elite male players compared with elite female players. More recently, Sarvestan, Svoboda [1] claimed that the volleyball players produce significantly more knee and hip extension angular velocities, take-off velocities, arm-swings, jump and spike heights, and impact velocities in successful spikes compared with faulty spikes. Despite this evidence base, it is still not clear how the multiple kinematic degrees of freedoms (DOF) are integrated to produce an accurate and efficient movement pattern in the volleyball spike.

According to dynamical systems theory, motor performance continually adapts to environmental and intrinsic constraints, organising the DOFs for efficient movement execution in a coordinated fashion [13]. In research carried out by Serrien, Ooijen [2] using self-organising maps (SOMs), an arbitrary Euclidean distance was employed to analyse coordination variability. Results demonstrated significantly higher coordination variability (a less stabile coordination patterning) in female volleyball spikers compared with the males. In a similar study investigating the proximal-to-distal coordination in young elite volleyball players using SOMs, Serrien, Goossens [14] showed that sex may be a large contributor to coordination variability, whilst maturation seemingly had no significant impact. SOMs, which are generally considered a class of artificial neural networks, are a concurrent approach being applied to investigate human movement [15]. Within the field of human movement sciences, these SOMs are adopted to explore complex movement patterns in sporting activities [2,16]. This machine learning-based approach could reduce dimensionality and aid in ease of interpretation of multiple-segment coordination patterning [16]. Currently, using the SOMs-based cluster analysis, Sarvestan, Svoboda [16] claimed that whole-body coordination patterning is an individually specific characteristic that remains relatively stable under different task constraints during volleyball spikes among attackers. 

Although an abundance of empirical investigations have analysed various kinematic aspects of the volleyball spike using various analytical methods, to date no study has been conducted to assess the whole-body coordination patterning in successful and faulty volleyball spikes. More detailed analytical methods would provide the necessary detail to accurately identify differences in a player’s body as a whole which would provide beneficial insight on overall performance. This could also help with sport-specific training, allowing coaches to design a multi-functional training program that targets multi-segment skill development as a whole. To this end, the main aim of this study was to investigate whole-body coordination patterning in successful and faulty spikes among young elite volleyball attackers using SOM-based cluster analysis. We hypothesised that there would be a significant difference between the coordination patterning of successful and faulty spikes.

## 2. Materials and Methods

### 2.1. Participants

A total of 13 young elite male attackers (Czech Republic national youth players) participated in this study, however only data for 10 players were included due to issues with data reconstruction. Table 1 demonstrates the general characteristics of included participants (*n* = 10). These consisted of six wing spikers, three middle blockers, and one opposite spiker. Across the participants, two were left-handed spikers (participant 2, wing spiker and participant 4, opposite spiker). Upon attending the laboratory, no acute injuries were reported by the attackers, and no players reported any musculoskeletal injuries or surgery within the last 12 months. Prior to any measures being taken, the purpose of the study and the risks of injuries were thoroughly explained to the participants, and both the players and their head coach signed informed consent forms.

### 2.2. Instrument and Procedure

Following a supervised 15-min dynamic warm-up by the researcher and coaches, the players performed six spikes as part of a sport-specific warm-up. A total of 37 retro-reflective, 14mm-diameter markers were attached to bony anatomical landmarks (head, C7, right scapula, T10, clavicle, sternal notch, acromion, upper arms, lateral humeral epicondyles, forearms, ulnar and radial styloid processes, anterior superior iliac spine, posterior superior iliac spine, thighs, lateral femoral epicondyle, tibia, Lateral malleoli, 1st metatarsal and heels) by a single researcher using the PlugInGait full-body model. Following marker placement, each attacker executed six spikes with the presence of two blockers from an individually chosen starting point. Six optoelectronic Vicon^®^ motion capture cameras (MX13+, Oxford Metrics, Oxford, UK) recorded the trajectories of all markers at a sampling frequency of 180 Hz. The global reference frames were defined as Z-axis (positive) in the upward direction, Y-axis in the anteroposterior direction (forward-positive) and X-axis in the mediolateral direction (right-positive).

Although the volleyball spike has formerly been investigated with the ball set in place using ropes at a specific location [11], we aimed to simulate real-game conditions by using an expert ‘setter’ to place the ball for the attackers. Therefore, the coach checked the accuracy of the setter and any inaccurate ‘set’ was repeated. Of the entire 60 sets, only one error, whereby the ball slipped from the setter’s hands, was observed and repeated. Furthermore, to check the within-subject impact location consistency, the locations of the wrist markers (ulnar and radial styloid process markers) were assessed at the moment of impact. 

### 2.3. Data Processing and Analysis

Data reconstruction and marker labelling was conducted using Vicon^®^ Nexus software (Version 1.8.6, Oxford Metrics, Oxford, UK). After filling any missed markers using spline and pattern methods (less than 10 missed frames), a 4th order Butterworth filter (0-lag) was used with a cut-off frequency of 10 Hz applied to smooth the trajectories and remove noise [17]. The corresponding static trial’s marker set was used to define joints, anatomically offset joint angles, and to locate each segment’s centre of mass [18,19]. Joint angles were calculated using the relative orientations of two adjacent segments (for flexion/extension, abduction/adduction, and external/internal rotation) [16]. Adopting the central difference method, the corresponding joint angular velocities were computed. Table 2 represents the kinematic variables used in the coordination patterning analyses. Note that both left and right limbs were incorporated into the analysis.

The time-series data of each spiking trial were first trimmed from the start of the plant phase to the moment of impacting the ball [20], which were then linearly interpolated to 101 data points. The plant phase was identified as the first frame that both feet made ground contact, and ball impact was identified as when the spiking hand wrist markers’ acceleration abruptly decreased in the Y-direction [16]. In this study, faulty spikes were defined as in Sarvestan, Svoboda [1], where the attacker was blocked, the spike velocity was lower than 50 Km·h^−1^, the ball touched the blocks and its velocity decreased to lower than 50 Km·h^−1^, or it touched outside of the area of play. The mean of successful and faulty spikes of individual participants was calculated and used for the SOM and cluster analysis. 

For the computation of whole body, lower limb, and upper limb coordination patterning, as well as joint range of motions (ROM) and angular velocities, we adopted SOMs [21]. The SOM, on the whole, is a 2D grid of weighted units, which are the prototype patterns of the input vectors. In this study, the adopted input vectors are a collection of kinematic variables at each time point:

(1) vi(t) = [ψi,1(t) … ψi,32(t) φi,1(t) … φi,32(t)]t

where ψk and φk, respectively, portray the degrees of freedom and the corresponding velocities (k = 1, …, 32; see Table 2). The i index is representative of the mean of all participants’ spikes (i = 1, …, 19); the number of spikes, i.e., 19 was determined as two trials per participant, minus faulty spikes for participant 2 (who did not fault during data collection). Prior to training the SOMs, the input vectors were normalised to −1 to 1 range intervals in order to unify the large kinematic differences between participants [21]. Thereafter, using competition and cooperation across the weight vectors, the SOMs iteratively updated the conversely stabilised solution via a self-organising process [21]. This process involved the Gaussian neighbourhood, hexagonal lattice, and sequential training types in a big map size. Table 3 summarises the parameters applied to the SOM and cluster analyses in this study. 

The adopted training method resulted in a hexagonal grid of units with every two neighbored hexagons having the most coordination state similarities. Since the SOM training adopts the entire successful and faulty spike performances, every two SOM panels demonstrate unified distance matrix (U-matrix) in whole body, lower, and upper limbs. Then, to detect the best-matching unit (BMU), the weight vectors with the smallest Euclidean distances were identified and unified. In the final step of the SOM, a pair-wise distance matrix was composed from the average of the entire coordination patterns by the trained SOM:

(2) $$D_{i,j}=\sum_{t=0}^{100}{\left[{\mathrm{BMU}}_{i}\left(t\right)-{\mathrm{BMU}}_{j}\left(t\right)\right]}^{2},\;i,\;j=1,\;\ldots ,\;19.$$

In the current study, the average data of the successful and faulty spikes were used for every player, except participant 2 as mentioned. Since they recorded only one faulty spike and the data of his faulty spike was removed in post-processing analysis of SOMs (due to technical complications), we used the average of all of their five successful trials.

In order to analyse the inter-individual coordination patterning of joint ROM and angular velocities in both successful and faulty spikes, we used cluster analysis on the matrixes derived from the SOMs. The ‘average linkage algorithm’ (see Table 3) was used to construct the hierarchical agglomerative clustering for every SOM. Thereafter, a dendrogram was created to represent the mean of trials per participant. Although we applied the cluster analysis for every SOM, only those with considerable differences from whole-body coordination patenting are presented in the results. All data processing and analysis, including the data reduction, interpolation, SOM analysis (SOM Toolbox) and clustering, were conducted using MATLAB software (Version 2020a, MathWorks, Inc., Natick, MA, USA). 

### 2.4. Statistical Analysis 

Since this study adopted an exploratory analysis approach where there were observable differences between the coordination, ROM, and angular velocity patterning, the spm1d statistical package (v0.4.3) (www.spm1d.org accessed on 10 February 2021) was used to identify significant differences of BMU trajectories between successful and faulty spikes. Following assessments of normality of data distribution using a Shapiro-Wilk’s test, the independent sample T-test (1d_ttest) was employed and significant differences were reported where observed. An alpha value of 0.05 was set a-priori. 

## 3. Results

**Figure 1:**
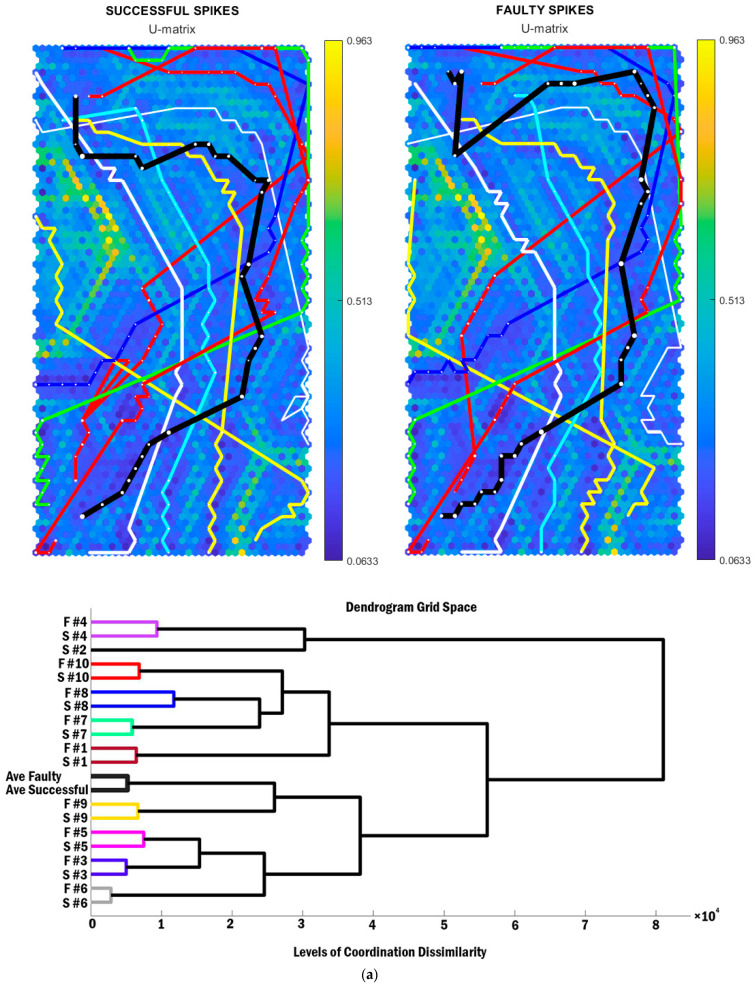

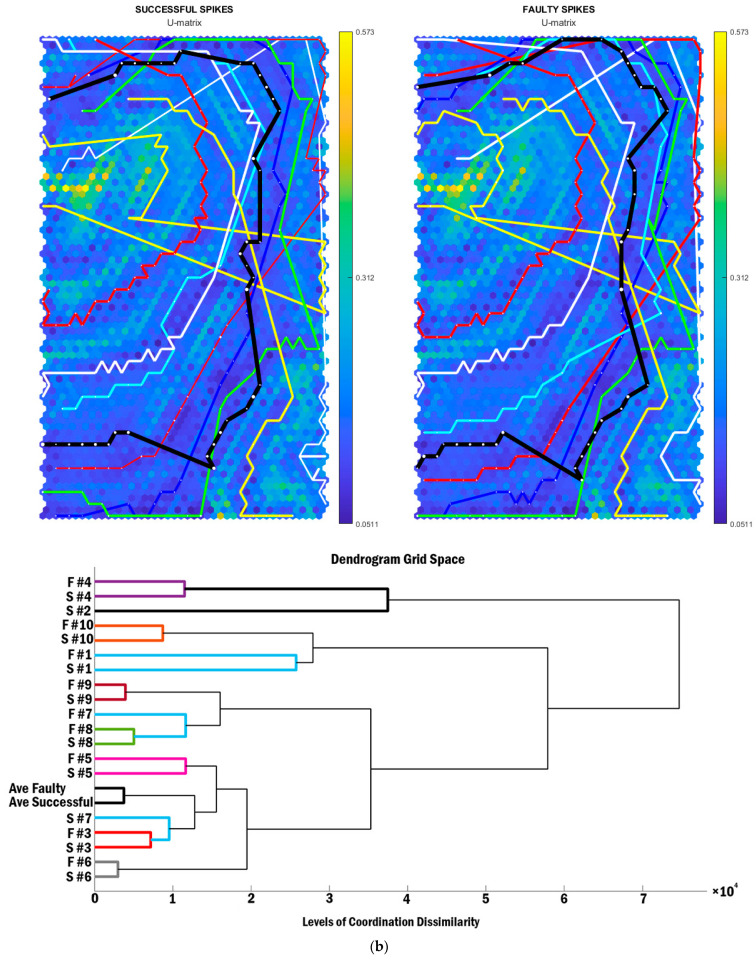

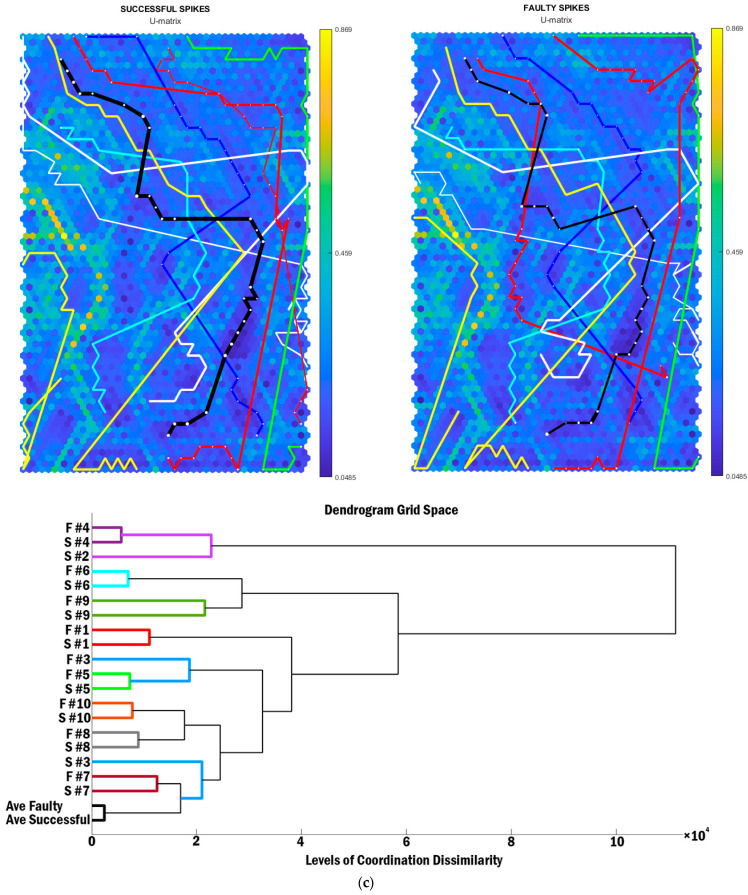
SOMs and cluster analysis of (**a**) whole-body coordination pattern, (**b**) lower limb coordination pattern, and (**c**) upper limb coordination patterning among the attackers. BMU trajectories of individual attackers (identified as a single-coloured line per individual) and the mean coordination patterning (black lines, or trajectories) in successful and faulty spike performances in each SOM. The orange-to-yellow colour of the hexagonal background depicts large Euclidean distance, while the blue colour depicts a small Euclidean distance.

Within-subject consistency impact locations were checked, and the maximum differences were 10 cm in the mediolateral direction, 16 cm in anteroposterior and 7 cm in the vertical direction. The SOMs, individual BMU trajectories, and cluster analysis of whole-body coordination patterning are depicted in Figure 1. Each hexagonal cell in the U-matrix indicates the Euclidian distance between the neighbouring SOM units. The colour of each unit also represents the average distance between the surrounding neighbor SOM units. The neighbours with fewer distances (blue cells) are also separated by the highly distanced cells (yellow cells). The green ridges have greater neighbouring distances than blue ridges and less neighbouring distances than yellow ridges. In this analysis, we depicted individual BMU trajectories in different colours (but matched in faulty and successful trials) to identify the similarities of successful and faulty pairs for each attacker. The average coordination patterning of successful and faulty spikes for all participants are presented in black BMU trajectories. Every BMU trajectory begins with the plant phase at the top and finishes with ball impact in the bottom of the SOMs.

The panels of Figure 1a identify two big differences on the left and bottom-right edges of the SOMs, specifically where the average coordination patterning is close to the yellow ridges (representing larger neighbouring distances). This portrays that the coordination patterning of successful and faulty spikes is particularly different at the initiation of spike performance where the participant starts to jump. Each pattern that is distinctly placed inside each of these two parts is significantly different from other patterns within the two ridges (near centre). Accordingly, only the initiation of the movement (the plant phase before jumping) and final phase (the cocking phase to impact event) for participants 8 are shown to be different from all other coordination patterning trajectories of attackers (Figure 1a, yellow line from the middle-left edge to the bottom-right edge). Individual BMU trajectories (Figure 1) revealed that every participant has a unique coordination pattern, both in successful and faulty spikes. However, there are differences between the whole-body coordination patterning of successful and faulty spikes between every attacker, particularly in the early and late phases of the volleyball spike. On the whole, the average BMU trajectories, both for successful and faulty spikes, demonstrate different coordination patterning of the whole body in the middle of the movement (Figure 1a, black line in top-right edge). Nevertheless, hierarchical clustering of the coordination patterning in SOMs confirms the similarities between the observed BMU trajectories of successful and faulty spikes between individual attackers. A unique coordination patterning was assigned to each attacker, whereby the BMU trajectories in successful spikes had the most similarities with the faulty trials of a similar attacker. Across individuals, participant 6 had the most similar coordination patterning between successful and faulty spikes (Figure 1a–c, green BMU trajectories), while participant 8 displayed the biggest differences in coordination patterning between successful and faulty trials (Figure 1a–c, white BMU trajectories). In addition, the coordination patterning of participants 2 and 4 was different to the rest of the attackers (levels of coordination dissimilarity > 8 × 104), while participants 3 and 5 had the most similar coordination patterning (levels of coordination dissimilarity < 2 × 104).

As Figure 1b represents, the mean lower limb coordinating patterning (black trajectories) was almost identical when comparing successful and faulty trials. However, this patterning was different between the successful and faulty trials of participant 7 (cyan trajectories). This was also identified by the cluster analysis, where the lower limb coordination patterning of the successful and faulty trials had the least similarities with other attackers.

**Figure 2:**
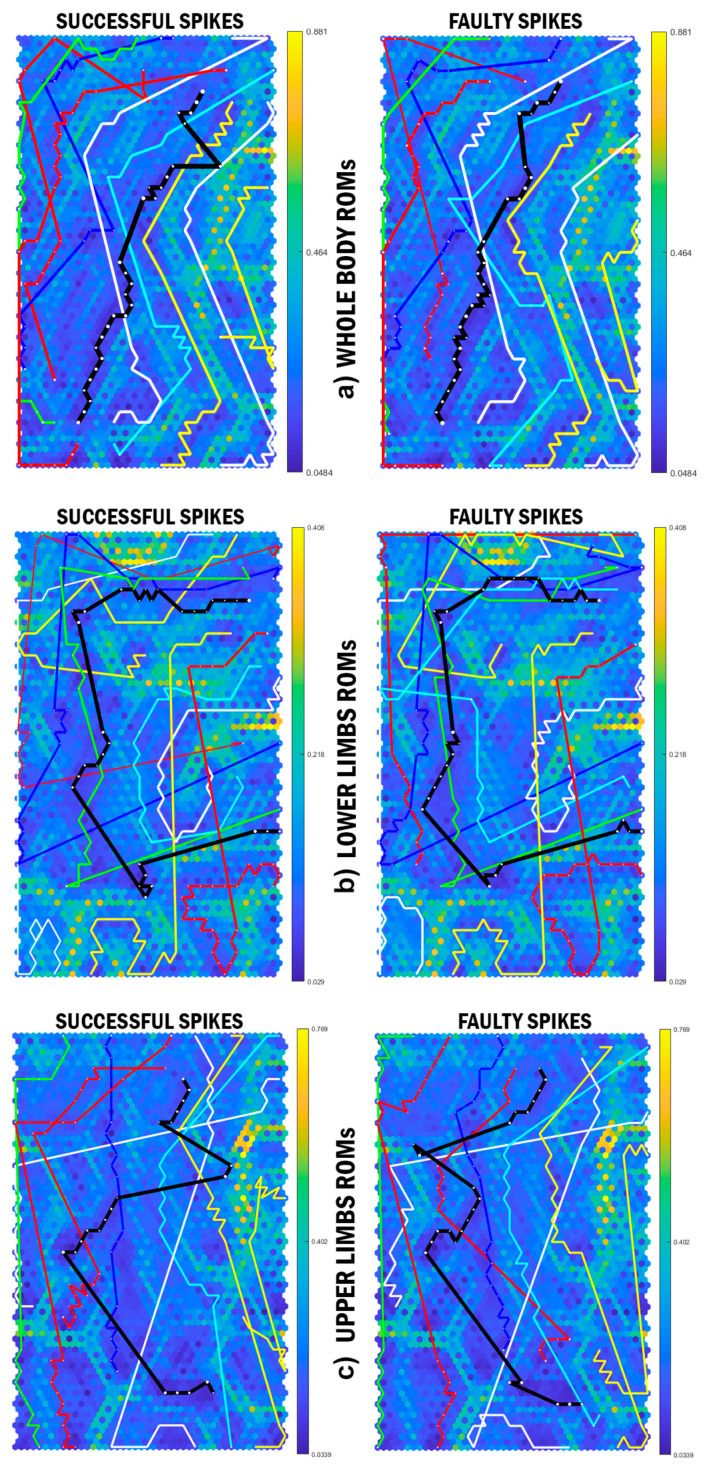
SOMs of (**a**) whole body, (**b**) lower limb, and (**c**) upper limb range of motions (ROM) of the attackers. BMU trajectories of individual attackers (identified as a single-coloured line per individual) and the mean coordination patterning (black lines, or trajectories) in successful and faulty spike performances in each SOM. The orange-to-yellow colour of the hexagonal background depicts large Euclidean distance, while the blue colour depicts a small Euclidean distance.

**Figure 3:**
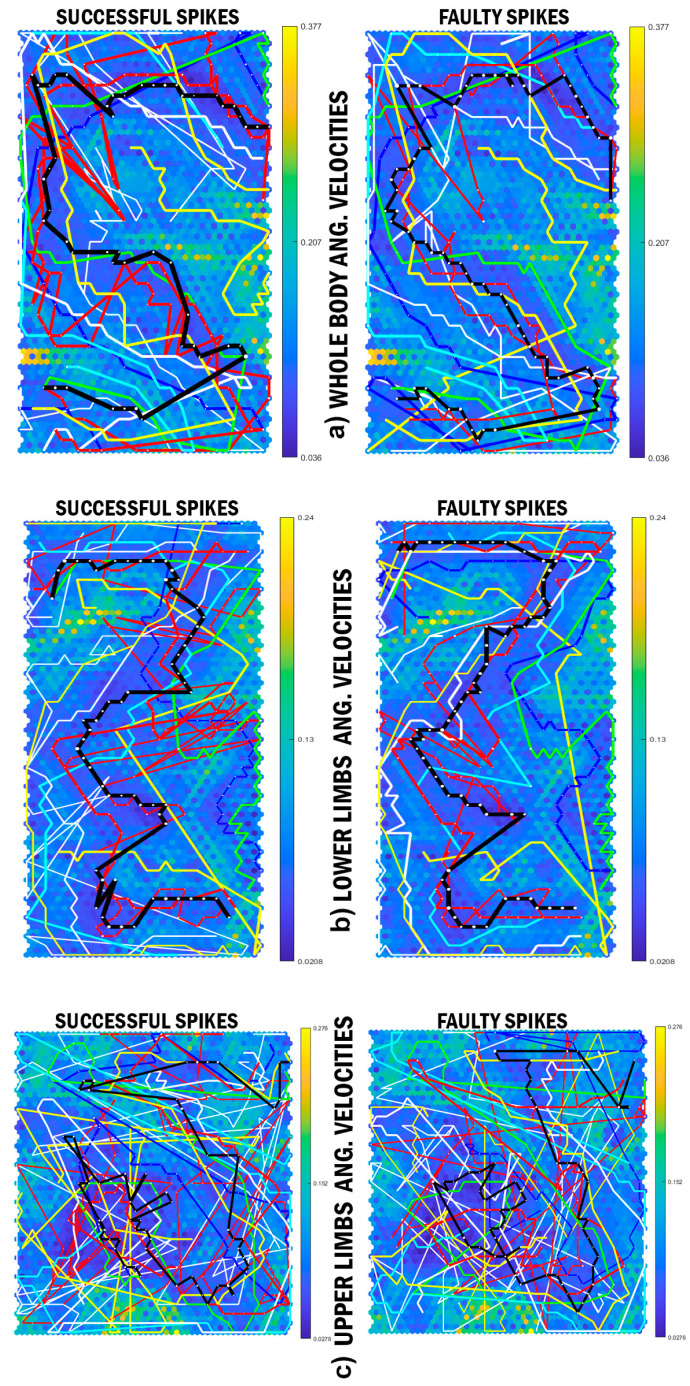
The SOMs of (**a**) whole body, (**b**) lower limbs, and (**c**) upper limb angular velocities among the attackers. BMU trajectories of individual attackers (identified as a single-coloured line per individual) and the mean coordination patterning (black lines, or trajectories) in successful and faulty spike performances in each SOM. The orange-to-yellow colour of the hexagonal background depicts large Euclidean distance, while the blue colour depicts a small Euclidean distance.

Analysis for the whole-body and lower limb ROM illustrated similar mean pattern for all attackers. In addition, the cluster analysis revealed similar results to the whole-body and upper limb coordination patterning across individuals (Figure 2a–c). Nevertheless, there are considerable differences between the average ROM patterns of upper limbs during the jump phase, where the black trajectories moved toward yellow ridges with high neighbouring distances in successful spikes. The cluster analysis of upper limb ROM was similar to the upper limb coordination pattern. A different pattern, however, is demonstrated with the upper-limb joint angular velocities (Figure 3c). Furthermore, although the whole-body and lower limb joint angular velocities are shown to have relatively different patterns, the cluster analysis depicted similar results as the patterning (Figure 3a,b).

**Figure 4:**
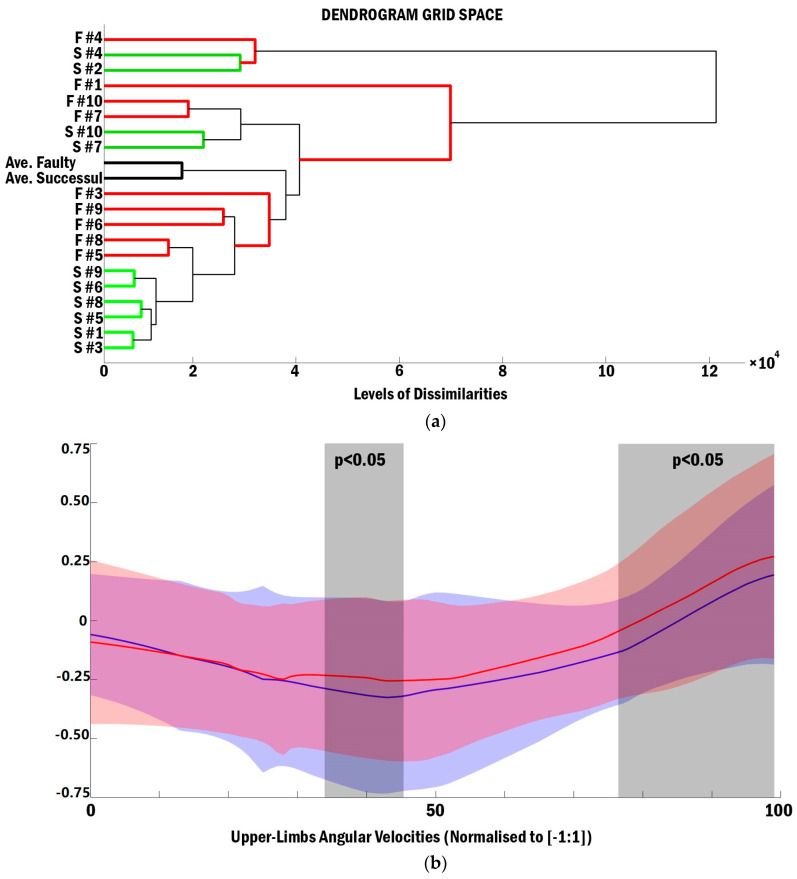
(**a**) Cluster analysis of the upper limb joint angular velocities in successful (green lines) and faulty (red lines) spikes, and (**b**) the SPM analysis portrays significant differences between the upper limb angular velocities in successful (red line) and faulty spikes (blue line) from 35% to 45% and from 76% to 100% of the spike movement (dark grey boxes).

The SPM analysis showed no significant differences between the whole body and lower limb joint angular velocities throughout the spike performance. In contrast, the upper limb angular velocities were significantly different between successful and faulty spikes, from 35% to 45% and 76% to 100% of the spike performance (Figure 4b). The cluster and SPM analyses confirmed this with the inter-individual similarities between the upper limb angular velocities in successful and faulty spikes (Figure 4a). Although the average upper limb angular velocities pattern of successful and faulty spikes remained similar, the dissimilarity levels were considerably high compared with the other average pattern (levels of coordination dissimilarity = 2 × 104).

## 4. Discussion

The aim of this research was to analyse the whole body coordination pattern differences between successful and faulty spikes in young elite volleyball attackers using SOM-based cluster analysis. The major finding of this study was that the upper limb angular velocities (using SOM and cluster-based analysis) were the main contributor to successful spikes among the attackers. The outcomes also showed that no inter-individual specific pattern exists when assessing whole body, lower and upper limb coordination patterning of successful and faulty spikes. Therefore, considerations on the level of analysis are important and likely related to the skill being assessed. It was also demonstrated that the upper limb angular velocities, using SOM and cluster-based analysis, were the main identifier between successful and faulty spiker coordination patterning among the attackers.

Adaptable movement in human performances is facilitated by the redundant degrees of freedoms (DOF) [22]. The CNS consistently constrains and modifies these DOFs using external (environmental) constraints and current spatiotemporal circumstances of the segment to find an optimal solution to the task [23]. The more skilled an individual becomes, the greater the integration of kinematic elements to aid in a coordinated, smooth performance by unifying the DOFs [24]. This increment in DOFs results in an individually unique movement pattern within athletes. The primary, and most important, observation of this study was that the whole body coordination patterning (joint ROM and angular velocities of whole body and centre of mass spatiotemporal characteristics) for each individual is unique. The cluster analysis also confirmed that regardless of success or error in the final result, the whole body pattern remains similar for each attacker. These observations reinforce the notion that the CNS increases the automaticity of the movement by limiting the DOFs to optimise the movement into a stable coordination pattern, allowing the working memory to be free to efficiently respond to further environmental disturbances [25]. The success or error of skill outcome, therefore, is linked to more fine elements of skill execution.

Similar to whole-body SOM and cluster analyses, a unique upper and lower limb coordination patterning was shown across all attackers, except the lower limb coordination patterning of participant 7 and upper limb coordination patterning of participant 3. Specific details in the average whole body coordination patterning reveals one large difference (the black trajectory on the top-right of the SOMs in Figure 2 and Figure 3) and several small differences (approximately the final 25% of the movement) between the successful and faulty spikes patterns. Previous kinematic data has demonstrated that joint angular velocity for the orientation leg, knee, hip, and also trunk were significantly larger in successful spike trials [1]. Higher Euclidean distances between successful and faulty spikes (since the green ridges have more neighbouring distances than blue ridges) also confirm the differences between the successful and faulty coordination patterns. To this end, it could be claimed that although the CNS increases the movement efficiency through controlling the DOFs, the angular velocities may be the main contributors to the success rates of the volleyball attackers. 

In the ROM analysis in our paper, the SOMs and BMU trajectories portrayed almost identical patterns in whole body and lower limbs; however, there was a visible difference between the upper limb ROM patterns in successful and faulty spikes. Further cluster and SPM analyses confirmed no significant differences between the ROM patterns of the whole body joint ROM in both successful and faulty spikes. It could be postulated, therefore, that skilled athletes model a complete linkage of the desired spike movement with defined DOFs and that the working memory automatically runs this model as a whole.

Interestingly, the SOM analysis demonstrated considerable differences across the individual angular velocities of the upper limb joints. Nevertheless, the mean angular velocity patterns were similar in the whole body and lower limb data (black lines). Conducting the cluster analysis, it was observed that the upper limb angular velocities were similar between the successful and faulty spikes of most of the attackers. In the left-handed attackers (participant 2 and 4, yellow and red lines in Figure 1, Figure 2 and Figure 3, respectively), the upper limb angular velocities of successful trials were more similar to each other. Among participants 1, 3, and 5–10, who were right-handed attackers, the upper limb angular velocities of successful/faulty trials were more similar (Figure 4b). The SPM analysis, in line with these findings, demonstrated significant differences between the upper limb angular velocities of the successful and faulty spikes around take-off (35–45% of the spike performance) and the last 25% of the movement (from where the attackers accelerate their hands to hit the ball). Kinematic results demonstrate that trunk and arm swing velocities at the take-off moment and the wrist angular velocities at impact were significantly higher in successful spikes [1]. 

Generally, in volleyball the main objective of an attacker is to achieve the greatest possible height to benefit from a larger field size for ball placement [4,26]. Therefore, a plethora of research focuses on increasing jump height through assessing lower limb explosive strength or coordination. The results of this study, in contrast, emphasise that the lower limb total coordination patterning might not be the primary element dictating the success rate of the volleyball spike, but rather the upper limb angular velocities. To this end, the majority of volleyball players and trainers place a premium on lower limb strength and capabilities during the volleyball spike, therefore, attackers have excellent lower limb capacities, but the upper limb capacities can be relatively untrained and thus lead to imbalances during skill execution. The result could either be faulty attacking performance or, more long-term, the potential for overuse injuries. Therefore, it is recommended that training focus on upper limb performance to improve volleyball spike performance.

### Limitations

Since the SOM analysis assesses global coordination patterns, ROM, and angular velocities, this level of analysis is not capable of presenting specific joint differences between successful and faulty spike performance. Nevertheless, the primary aim of this study was to analyse the attackers’ performance on a global level, therefore the results of this study provide a unique contribution. 

## 5. Conclusions

The SOMs-based cluster analysis, as a class of artificial neural networks, was shown to be an appropriate tool for analysing and identifying whole body coordination pattern differences between successful and faulty spikes in elite volleyball attackers. Findings indicated that regardless of success or error in the outcome of a volleyball spike, whole body coordination patterning is unique for each participant. The CNS strictly governs the entire body and joint ROM to control redundancy of the DOF and maintain coordination. Analysis of the upper limb angular velocity demonstrates differences between successful and faulty spikes within the individual attackers. To this end, despite previous research identifying that the lower limbs are the main contributor to successful spike performance, the upper limbs seemingly play a pivotal role in the success rate of the volleyball spike. To this end, it is recommended that volleyball coaches design their training programs to focus on the upper extremities, and also to focus on limbs as a whole rather than individual segments. The SOM analysis was also shown to be a useful tool for the evaluation of several segments on a global level and provides practitioners with useful complex skill execution measures. 

## Figures and Tables

**Table 1:** General characteristics of the participants.

Variable	Mean ± SD
Age (year)	15.5 ± 0.7
Height (cm)	192.9 ± 4.1
Weight (Kg)	76.9 ± 4.7
Experience (year)	6.9 ± 0.7

**Table 2:** Overview of kinematic variables used in the self-organising map (SOM) coordination profile analysis.

Joint/Segment	Abbreviations	Descriptions
centre-of-mass	CoMX, CoMY, CoMZ	3D coordinates of the total-body centre of mass
Left and right lower limb joints	LhipX, LkneeX, LankleX RhipX, RkneeX, RankleX	Flexion-extension angles of the hip, knee, and ankle joints
Left and right upper limb joints	LshoulderX, LshoulderY, LshoulderZ, LelbowX RshoulderX, RshoulderY, RshoulderZ, RelbowX	Shoulder ab/adduction, horizontal ab/adduction and internal/external rotation, elbow flexion-extension
Trunk	PelvisX, PelvisY, PelvisZ SpineX, SpineY, SpineZ ThoraxX, ThoraxY, ThoraxZ	Pelvis tilt, obliquity, and rotation (absolute) Spine flexion/extension, lateral flexion, and rotation (relative) Thorax tilt, obliquity, and rotation (absolute)
Neck and head	NeckX, NeckY, NeckZ HeadX, HeadY, HeadZ	Neck flexion/extension, lateral flexion, and rotation (relative) Head tilt, obliquity, and rotation (absolute)

**Table 3:** Parameters explored in the sensitivity analysis. Applied options in the result section of this study are in bold.

SOM Parameters		Options						
**Map size**		Small (¼× default)		Normal (default)			**Big (4× default)**	
**Neighbourhood**		**Gaussian**		Cut-off Gaussian			Bubble	Epanechicov
**Lattice**		**Hexagonal**		Rectangular				
**Training type**		**Sequential**		Batch				
**Cluster Linkage Algorithm**								
Single	Complete		**Average**		Median	Centroid		Ward’s

## Data Availability

The data of the present manuscript is available on demand from the corresponding author.
